# Effect of Copper-Modified ZSM-5 Zeolite Concentration on the Thermomechanical and Antimicrobial Properties of Compatibilized Native Starch/Polylactic Acid Blends

**DOI:** 10.3390/polym18172036

**Published:** 2026-08-22

**Authors:** Karla Garrido-Miranda, Elizabeth Moreno-Bohorquez, Mary Judith Arias-Tapia, Cristian Miranda, Ángelo Oñate, Carlos Lanziotti, Ángel Contreras, Jesús D. Rhenals-Julio, Andrés F. Jaramillo, Manuel F. Melendrez

**Affiliations:** 1 Scientific and Technological Bioresource Nucleus, BIOREN, Universidad de La Frontera, Temuco 4811230, Chile; 2 Chemical Engineering Program, Faculty of Engineering, Universidad Tecnológica de Bolívar, Parque Industrial and Tecnológico Carlos Vélez Pombo km 1 Vía Turbaco, Cartagena de Indias 130001, Colombia; morenoe@utb.edu.co (E.M.-B.); mariast@utb.edu.co (M.J.A.-T.); 3 Unidad de Desarrollo Tecnológico, 2634 Av. Cordillera, Concepción 4191996, Chile; c.miranda@udt.cl; 4 Department of Materials Engineering (DIMAT), Faculty of Engineering, Universidad de Concepción, 315 Edmundo Larenas, Concepción 4070415, Chile; aonates@udec.cl; 5 Department of Mechanical Engineering (DIM), Faculty of Engineering, University of Concepción, Edmundo Larenas 219, Concepción 4070409, Chile; clanziotti@udec.cl; 6 Departamento de Ciencias Biológicas y Químicas, Universidad Católica de Temuco, Temuco 4813302, Chile; acontrer@uct.cl; 7 Departamento de Ingeniería Mecánica, Universidad de Córdoba, Cr 6 #76-103, Montería 230002, Colombia; jesusrhenalsj@correo.unicordoba.edu.co; 8 Department of Mechanical Engineering, Universidad de La Frontera, Temuco 4780000, Chile; 9 Facultad de Ingeniería, Universidad San Sebastián, Campus Las Tres Pascualas, Lientur 1457, Concepción 4060000, Chile; manuel.melendrez@uss.cl

**Keywords:** biodegradable polymer composites, copper-modified zeolite (ZSM-5), thermomechanical properties, antimicrobial activity, citric acid crosslinking

## Abstract

The development of multifunctional biodegradable materials with improved structural performance and antimicrobial functionality is essential for advancing sustainable packaging. This study evaluates the effect of copper-modified ZSM-5 zeolite (ZCu) concentration (0, 1, and 5 wt%) on thermoplastic starch/polylactic acid (TPS/PLA) blends. The TPS was derived from *Ipomoea batatas* (sweet potato, SP) and *Dioscorea rotundata* (diamond yam, DY) starches and compatibilized with 1 wt% citric acid. The ZCu response depended on both ZCu loading and the botanical starch source. Structural analyses showed that SP-based composites reached their highest crystallinity at 1 wt% ZCu (13.70%), whereas DY-based systems exhibited an initial decrease at 1 wt% followed by an increase to 12.75% at 5 wt%, reflecting distinct concentration-dependent crystallization trends. Thermal analyses demonstrated a substantial increase in the degradation onset temperature of SP-based composites, from 132.9 °C to 185.1 °C at 1 wt% ZCu, indicating an effective thermal barrier effect. Nanomechanical mapping revealed concentration- and starch-source-dependent changes in local hardness, reduced modulus, and elastic recovery, without evidence of uniform mechanical reinforcement across all formulations. Antibacterial activity was observed exclusively in composites containing 5 wt% ZCu, with inhibition zones of 5.3 mm against *Staphylococcus aureus* and 1.0 mm against *Escherichia coli*. These findings highlight how the structural, thermal, nanomechanical and antimicrobial responses of TPS/PLA blends vary with ZCu concentration and the botanical origin of the starch.

## 1. Introduction

The growing demand for sustainable materials suitable for advanced manufacturing and food preservation applications has intensified research on biodegradable polymer composites [1]. Biodegradable polymers are increasingly regarded as promising alternatives to conventional polymers; however, their broader implementation is often constrained by comparatively lower mechanical performance, thermal resistance, and barrier properties [2]. To overcome these limitations, several modification strategies have been explored, including reinforcement with natural fibers, nanoparticles, and inorganic fillers, which can improve the structural and functional performance of biodegradable polymer matrices [2]. Among their potential applications, food packaging is particularly relevant because of the environmental persistence and accumulation of conventional packaging waste. Nevertheless, the development of biodegradable materials for food packaging requires a careful balance between sustainability and functional performance. In addition to adequate mechanical integrity and thermal stability, advanced packaging materials may benefit from active functionalities, particularly antimicrobial activity, to inhibit microbial growth, enhance food safety, and potentially extend product shelf life [2].

Inorganic fillers can modify several functions of biodegradable matrices through their surface chemistry, thermal stability and interaction with polymer chains [3]. Zeolites are particularly attractive because their crystalline aluminosilicate frameworks provide high thermal stability, ion-exchange capacity and microporosity, while zeolite particles may act as rigid fillers or heterogeneous nucleation sites [4,5]. ZSM-5 is a microporous crystalline aluminosilicate with an MFI framework. Modification with copper can introduce antibacterial functionality through the release of Cu species and the regeneration of reactive oxygen species, while retaining the zeolite framework. The copper-modified ZSM-5 formulation used here was previously characterized and showed antimicrobial activity against *Idiomarina loihiensis*, *Pseudoalteromonas*, and *Halomonas boliviensis* with inhibition zones of 50.7–61.0 mm [6,7].

Among biobased systems, thermoplastic starch/polylactic acid (TPS/PLA) blends have emerged with promising potential due to its renewable origin, biodegradability, and tunable properties [8]. Their main limitation is the low compatibility between hydrophilic starch and comparatively hydrophobic PLA, which can produce phase separation and weak interfacial adhesion [9]. Citric acid-mediated reactive compatibilization has therefore been previously used to improve interfacial continuity and the thermal and mechanical response of TPS/PLA blends [10,11,12].

The response of a TPS/PLA matrix to an inorganic filler may also depend on the botanical source of starch. The sweet potato starch (*Ipomoea batatas*) contained 45.9% amylose, whereas the yam starch (*Dioscorea rotundata*) contained 25.59% amylose. Differences in the relative proportions of linear amylose and branched amylopectin, together with source-dependent granule and hydroxyl group organization, may affect plasticization, interfacial interactions, filler dispersion and crystallization behavior [4,13].

Moreover, even optimized TPS/PLA systems lack intrinsic antimicrobial activity and may require additional reinforcement to meet performance demands of active packaging and advanced manufacturing technologies [14]. In food-packaging applications, the absence of antimicrobial functionality limits their ability to extend product shelf life and ensure product safety, while insufficient stiffness and thermal resistance can compromise dimensional stability during processing and use.

Although the citric acid-compatibilized TPS/PLA matrices and the copper-modified ZSM-5 system were previously developed separately [7,14], their integration has not been comparatively evaluated. This distinction is important because the effect of a given filler loading may differ depending on the starch source. The contribution of this work is therefore the comparative evaluation of identical ZCu loadings in TPS/PLA matrices that differ only in the botanical origin and intrinsic characteristics of the starch. This design separates the effect of ZCu concentration from changes in the overall TPS/PLA composition and allows concentration-dependent trade-offs among structure, thermal stability, local nanomechanical response and antibacterial response to be identified.

Therefore, the aim of this study is to investigate the structural, thermomechanical, and antimicrobial effects of incorporating copper-modified ZSM-5 zeolite (0, 1 and 5 wt%) into a previously optimized TPS/PLA (60/40) matrix derived from *Dioscorea rotundata* (yam—DY) and *Ipomoea batatas* (sweet potato—SP), to elucidate how ZCu concentration and starch source influence the structural, thermal, nanomechanical and antimicrobial responses of compatibilized TPS/PLA composites.

## 2. Materials and Methods

### 2.1. Raw Materials

Synthetic ZSM-5 (840NHA) zeolite with compensating NH^4+^ cations was purchased from TOSOH (Grove City, OH, USA). Copper nitrate trihydrate (Cu[NO_3_]_2_⋅3H_2_O) (99.9% purity) and ethanol (C_2_H_6_O) (purity ≥ 99.5%) were supplied by Merck S.A. (Darmstadt, Germany). TPS/PLA/CA blends were obtained from a previously developed formulation reported in [14]. These base matrices were originally prepared from polylactic acid (PLA), native starch, citric acid as a compatibilizer, and glycerol as a plasticizer.

This study builds upon previous studies on TPS/PLA matrices compatibilized with citric acid [14] and copper-modified ZSM-5 zeolites with antimicrobial activity [7]. In this work, both approaches are integrated by incorporating ZCu into the optimized matrix. The factors evaluated were ZCu concentration and botanical starch source, while the TPS/PLA ratio and CA content were maintained at a constant level.

### 2.2. Synthesis of Copper-Modified ZSM-5 Zeolite

Copper incorporation into ZSM-5 was carried out following a previously reported wet impregnation procedure. Copper nitrate trihydrate was used as a precursor to obtain a nominal copper loading of 8 wt% relative to zeolite mass. This loading was selected based on prior studies evaluating different loadings (0–8 wt%) that demonstrated optimal antimicrobial performance without compromising the structural stability of the ZSM-5 framework [7].

The zeolite was suspended in the precursor solution under continuous stirring at 90 °C using a rotary evaporator (EV400H, LabTech, Sorisole, Italy) to promote diffusion of copper species into the pore network. Subsequent solvent removal under vacuum, followed by controlled thermal treatment (24 h at 100 °C) allowed the deposition of copper species within the zeolite structure. The modified zeolite was stored in a desiccator until further use. The ZCu formulation used in this study was selected based on its previously reported characterization and antimicrobial performance [7]. Therefore, its independent characterization was not repeated in the present work.

### 2.3. Preparation and Processing of TPS/PLA-Based Composites Reinforced with Copper-Modified ZSM-5 Zeolite

The TPSSP/PLA and TPSDY/PLA blends (60% TPS/ 40% PLA) compatibilized with citric acid-grafted starch (CA) at 0%, 1%, and 5% were obtained from a previously reported study by Martinez-Villadiego et al. [14]. In that work, native starches from *Ipomoea batatas* (SP) and *Dioscorea rotundata* (DY) was extracted, plasticized with glycerol to obtain thermoplastic starch (TPS), and subsequently melt-blended with polylactic acid (PLA) in the presence of citric acid as a compatibilizing agent (0–5 wt%). These materials were used as base matrices in the present investigation. Among the available formulations, the TPS/PLA blend compatibilized with 1 wt% compatibilizer was selected for composite fabrication based on its previously reported performance [8,14].

For composite fabrication, melt mixing was performed using a torque rheometer (model 835205, Brabender GmbH & Co. KG, Duisburg, Germany). Mixing was carried out for 7 min at 60 rpm at 140 °C. The composites were obtained by adding ZCu at concentrations of 0, 1 and 5 wt% to the TPS/PLA blend compatibilized with 1 wt% CA.

Finally, square plates (100 mm × 100 mm × 1 mm) were fabricated from each material to ensure sample homogeneity for subsequent analyses. The composites were compression molded using a Lab-Tech hydraulic press (model LP 20 B, Labtech Engineering Co., Ltd., Samut Prakan, Thailand) at 160 °C for 5 min under a pressure of 110 bar. Preheating and cooling times were 150 min and 1 min, respectively.

Samples were coded according to starch source and ZCu content as BM-SP/Zx and BM-DY/Zx, where BM denotes the base matrix, SP and DY the starch source, and x represents the ZCu content (0, 1, or 5 wt%).

### 2.4. Structural and Chemical Characterization

#### 2.4.1. X-Ray Diffraction (XRD)

X-ray diffraction (XRD) patterns were recorded using a Bruker Endeavor diffractometer (D4/MAX-B, Bruker, Billerica, MA, USA). The instrument was operated at 40 kV and 20 mA using Cu kα (λ = 1.541 Å). Data were collected over a range of 4–80°, with a step size of 0.02° and a counting time of 1 s per step.

The degree of crystallinity was determined from the XRD patterns using a numerical peak integration approach based on the methodology described by Podgorbunskikh et al. [15]. Following baseline subtraction, crystalline reflection peaks were identified and bounded through automated peak width analysis. The integrated areas of both the crystalline peaks and the broad amorphous background halo were calculated via direct trapezoidal numerical integration. The degree of crystallinity was quantified as the percentage ratio of the integrated crystalline peak area to the total diffraction area (sum of crystalline and amorphous regions).

#### 2.4.2. Fourier Transform Infrared Spectroscopy (FTIR)

FTIR analysis was performed using a PerkinElmer PECTRUM 2 infrared spectrometer (model 17203, PerkinElmer, Waltham, MA, USA) equipped with an attenuated total reflectance (ATR) accessory and a ZnSe crystal. Each spectrum was obtained from consecutive scans at a resolution of 4 cm^−1^.

### 2.5. Morphological Analysis (SEM)

Surface morphology was examined by scanning electron microscopy (SEM) using an SU-3500 microscope (Hitachi High-Tech Corporation, Tokyo, Japan) operated at an accelerating voltage of 10 kV under high-vacuum conditions (30 Pa). Micrographs were acquired at magnifications corresponding to scale bars ranging from 5 to 100 μm.

### 2.6. Thermal Properties

#### 2.6.1. Thermogravimetric Analysis (TGA)

Thermogravimetric analysis (TGA) was performed using a (NETZSCH-Gerätebau GmbH, Selb, Germany) TG209F3 instrument. Approximately 5 mg of sample was heated from 25 °C to 600 °C at a heating rate of 10 °C/min. Measurements were carried out in aluminum crucibles under a nitrogen atmosphere.

#### 2.6.2. Differential Scanning Calorimetry (DSC)

Differential scanning calorimetry (DSC) analyses were performed using a (NETZSCH-Gerätebau GmbH, Selb, Germany) instrument. Nanocomposite samples (5–10 mg) were placed in sealed aluminum crucibles. The measurements consisted of a heating–cooling–heating cycle. Initially, the samples were heated from 25 °C to 200 °C at a rate of 10 °C/min followed by controlled cooling and a second heating cycle under the same conditions. All measurements were conducted under a constant nitrogen flow of 20 mL/min.

### 2.7. Mechanical Properties

Instrumented nanoindentation experiments were performed using a Hysitron TI 980 TriboIndenter^®^ (Bruker, Billerica, MA, USA). The experimental conditions were established using a 9 s trapezoidal loading cycle consisting of 3 s loading, 3 s holding at the maximum load, and 3 s unloading. Measurements were conducted in Accelerated Property Mapping (XPM) mode under a constant maximum load of 100 µN, a data acquisition rate of 500 points s^−1^, and a lateral scanning speed of 0.5 µm s^−1^.

The analyses were carried out using a 5 × 5 indentation matrix (25 indentations in total), with a spacing of 1 µm between adjacent indentations in both the horizontal and vertical directions, corresponding to a mapped area of 4 µm × 4 µm based on the center-to-center distance between the outermost indentation points. A Berkovich diamond indenter was employed, considering a Poisson’s ratio of 0.07 and an elastic modulus of 1140 GPa for the indenter tip.

Prior to testing, the indenter tip was calibrated using a fused quartz reference specimen by performing indentations at a maximum load of 8000 µN. Subsequently, the indenter was positioned on the sample surface until the initial contact condition was achieved. Mechanical properties were determined according to the Oliver–Pharr method using the calibrated tip area function as a function of contact depth:

(1) $$A=f\left(h_{c}\right)$$

where (A) is the projected contact area and (h_c_) is the contact depth during indentation. In addition, the Elastic Recovery Index (ERI) was determined from the load–displacement curves obtained at each measurement point. This parameter quantifies the ability of the material to recover the deformation induced during the indentation cycle and was calculated according to

(2) $$ERI=\frac{h_{max}-h_{r}}{h_{max}}$$

where (h_max_) corresponds to the maximum penetration depth reached during loading and (h_r_) represents the residual depth after complete unloading. ERI values approaching unity indicate a predominantly elastic response with minimal permanent deformation, whereas lower values reflect a greater contribution of irreversible deformation mechanisms, including plastic deformation and viscoelastic relaxation.

The ERI values obtained at each indentation location were assigned to their corresponding spatial coordinates and subsequently used to generate two-dimensional distribution maps through interpolation of the experimental data. These maps enabled the evaluation of the surface mechanical homogeneity of the investigated polymers and the identification of local variations associated with microstructural heterogeneities, phase distribution, or differences in the crosslinking density of the polymer matrix.

### 2.8. Antimicrobial Activity

The antimicrobial activity of the prepared composites was evaluated against reference bacterial strains *Staphylococcus aureus* and *Escherichia coli*, following the Kirby–Bauer disk diffusion method. Bacterial suspensions were prepared from fresh pure cultures and adjusted to a concentration of 1.5 × 10^6^ UFC/mL (0.5 McFarland standard). Aliquots of 200 μL were uniformly spread onto agar plates and allowed to dry for 20 min under sterile conditions. Disks impregnated with the composite materials were then placed onto the inoculated surfaces and incubated at 35 ± 1 °C for 24–48 h. After incubation, inhibition zones surrounding each disk were measured and recorded. Control and experimental groups for both bacterial strains were tested in triplicate.

## 3. Results and Discussion

### 3.1. Structural and Chemical Characterization

#### 3.1.1. Crystalline Structure and Phase Analysis by X-Ray Diffraction (XRD)

**Figure 1:**
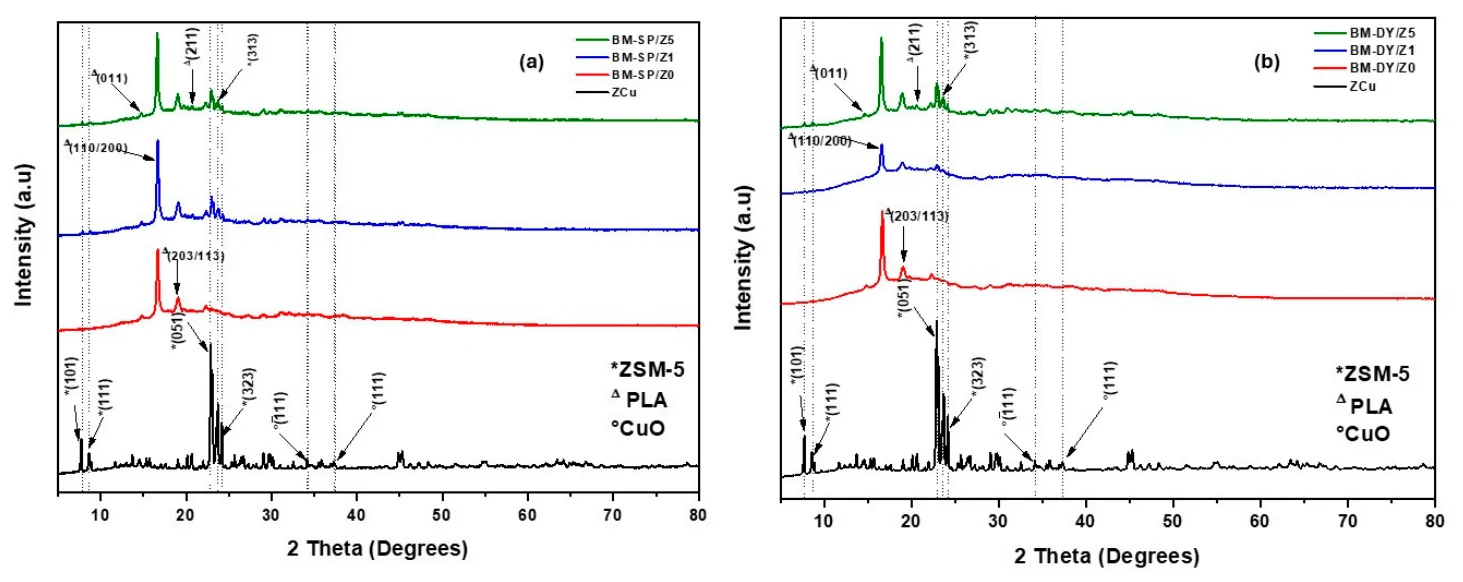
XRD diffractograms of (**a**) SP and (**b**) DY reinforced blends. The ZCu reference pattern was previously reported in [7].

XRD analysis was performed to study the crystalline behavior of the reinforced blends. The diffractograms of the blends containing SP and DY starch are illustrated in Figure 1. All formulations exhibited the characteristic α-PLA reflections at 16–17°, 19–20°, and ~22°, confirming that the crystalline structure of PLA is preserved after processing [16,17]. These reflections correspond to the α-PLA crystalline phase, particularly the (110/200), (203/113), (011), and (211) planes, as reported for α-PLA systems [18]. The amorphous halo between 10–25° is observed in all samples, associated with TPS and the amorphous PLA fraction [19], indicating effective starch disruption during plasticization. The preservation of these diffraction features, together with the amorphous halo, confirms that the polymeric matrix retains its semi-crystalline nature after processing and filler incorporation.

The estimated crystallinity values are presented in Table 1. For the SP-based composites, crystallinity changed from 12.09% for BM-SP/Z0 to 13.70% for BM-SP/Z1 and 13.07% for BM-SP/Z5. For the DY-based composites, the corresponding values were 10.73%, 9.94% and 12.75%, respectively. Because these values were obtained from single determinations and the differences among formulations were relatively small, they are interpreted as descriptive trends rather than statistically significant changes.

The different responses observed for the two matrices may be related, at least in part, to their different amylose contents. SP starch contained 45.9% amylose, whereas DY starch contained 25.59% [13]. This difference in the proportion of linear and branched chains may influence matrix organization and matrix–filler interactions. Previous studies have shown that amylose/amylopectin ratio can influence crystalline organization, polymer chain packing and filler dispersion. However, the effect may also depend on the reinforcement geometry [20,21].

Additional reflections at 6–8° and 23–25° were observed in the reinforced composites and were assigned to the characteristic MFI framework of ZSM-5. These reflections were absent in the unfilled matrices, suggesting that the zeolite crystalline structure was preserved after composite processing [22]. This behavior is consistent with the findings of Huenuvil-Pacheco et al. [7], which determined via X-ray diffraction (XRD) that the MFI framework of ZSM-5 zeolite remains stable after modification with copper. The CuO-related reflections previously reported for the ZCu material were not clearly resolved in the composite patterns, possibly because of the low ZCu content and overlap with the polymer matrix.

The higher peak intensity observed for BM-DY/Z5 reflects the greater contribution of the crystalline inorganic phase at this ZCu concentration.

#### 3.1.2. Fourier Transform Infrared (FTIR) Spectroscopy

**Figure 2:**
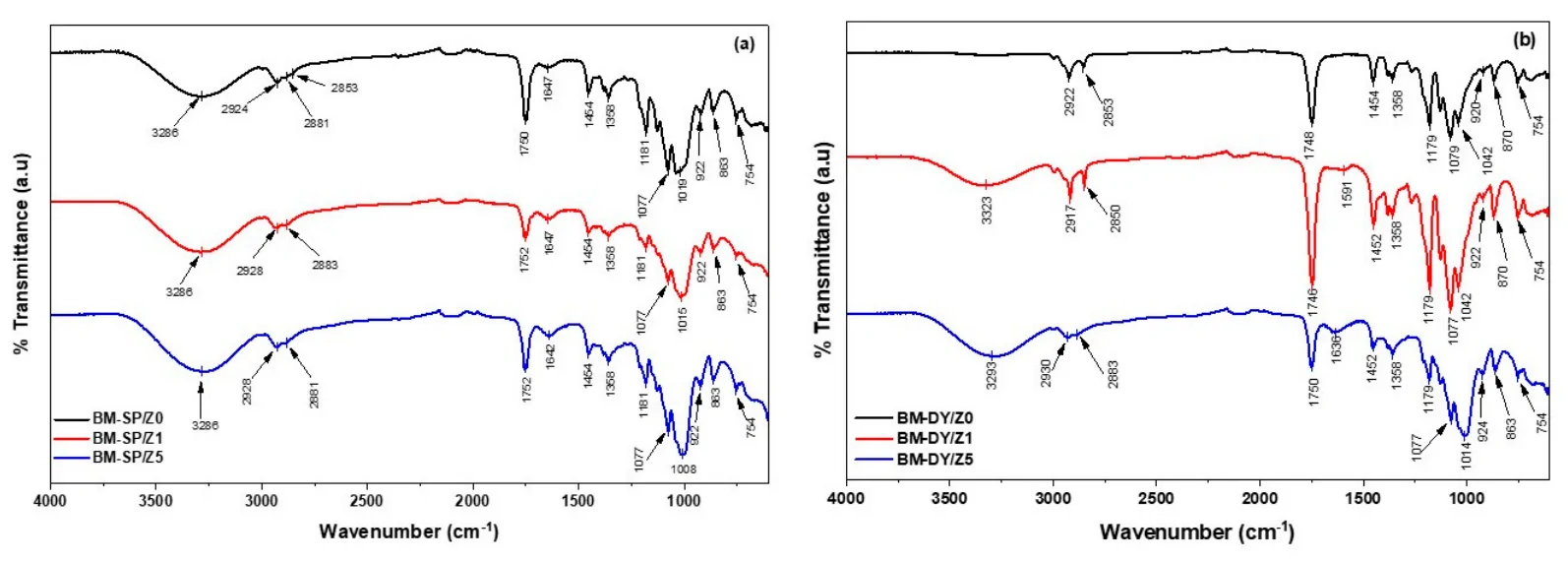
FTIR spectra of (**a**) SP- and (**b**) DY-based composites.

FTIR spectra for SP- and DY-based composites are shown in Figure 2. All formulations exhibited the characteristic PLA carbonyl band at approximately 1750 cm^−1^ [23]. Additional bands were observed at ~1452 cm^−1^ (-CH_3_ stretching), ~1185 cm^−1^ (C-O-C stretching), between 863 and 758 cm^−1^ (-C-C- stretching of amorphous/crystalline phases) [24], and approximately 3300 cm^−1^, corresponding to -OH groups from the starch-rich phase [14].

Despite the different botanical origins and amylose contents of the SP and DY starches, both composite systems exhibited the same principal functional groups. This indicates that the different starch sources did not result in additional resolved bands attributable to new chemical functionalities, although it may influence molecular organization and hydrogen-bonding interactions within the matrix. These possible differences were not quantitatively assessed because the spectra were not normalized.

The characteristic vibrations of the MFI framework occur mainly within the 450–1250 cm^−1^ region and overlap with the intense absorption bands of the polymer matrix. Consequently, separate contributions from the zeolite framework were not clearly resolved in the composite spectra. No additional resolved bands were observed after ZCu incorporation.

### 3.2. Morphological Characterization and Filler Dispersion Analysis by SEM

**Figure 3:**
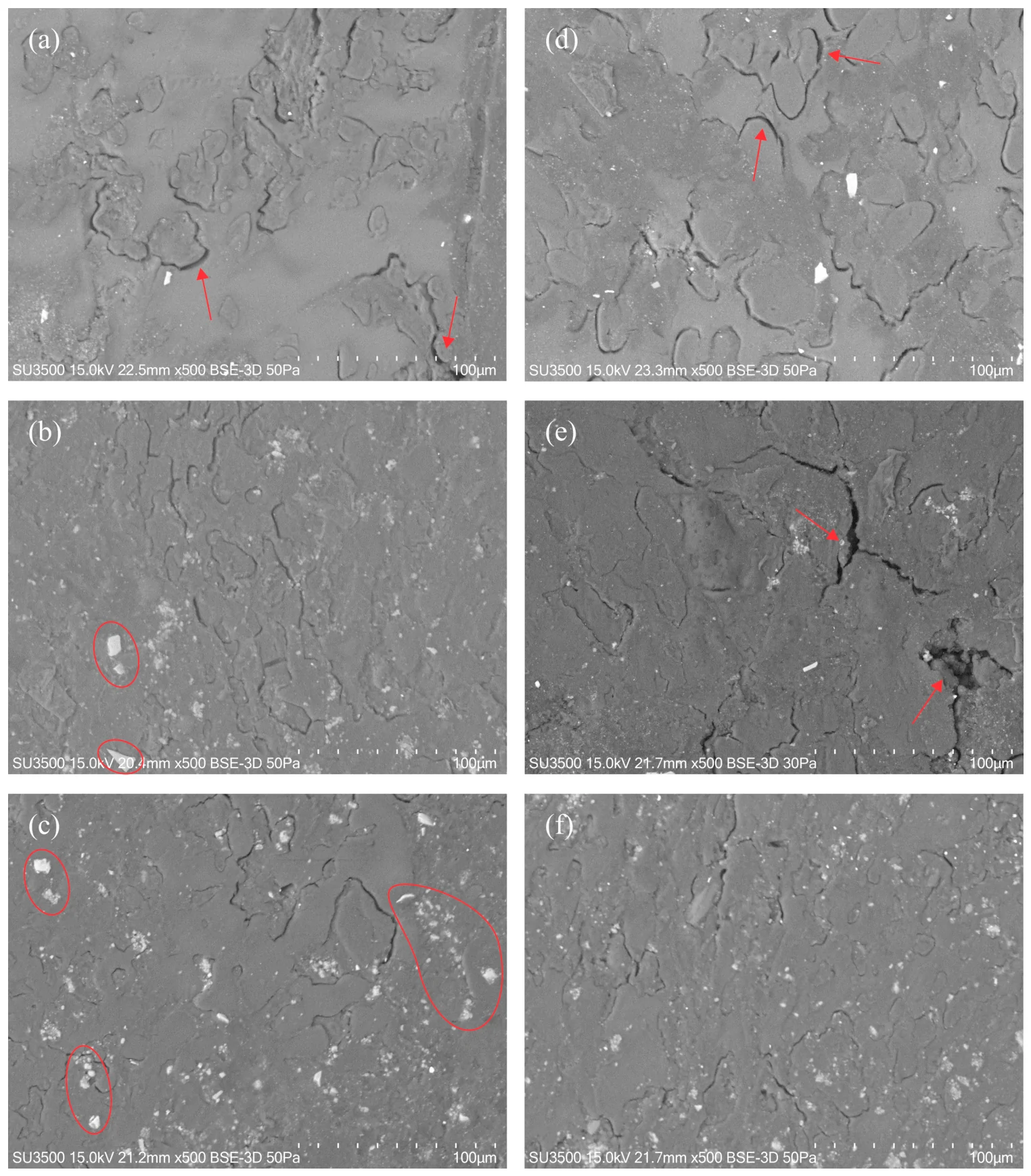
SEM micrographs of (**a**–**c**) SP- and (**d**–**f**) DY-based composites reinforced with 0, 1, and 5% copper-modified zeolite at 500×.

The morphology of the blends was studied through SEM. The cross-sectional micrographs of the SP- and DY-based blends are shown in Figure 3. BSE mode was employed to assess zeolite dispersion based on the appearance of bright domains of the inorganic phase.

In SP-based systems, BM-SP/Z0 (Figure 3a) exhibited a relatively homogeneous matrix with irregular boundaries and occasional interfacial discontinuities, indicating that some TPS/PLA phase heterogeneity remained after compatibilization. At 1 wt% ZCu (Figure 3b), small bright domains were broadly distributed throughout the matrix, with no extensive particle agglomeration. At 5 wt% (Figure 3c), the density of bright domains increased and localized clusters were observed, indicating a less homogeneous filler distribution.

In the DY-based systems, BM-DY/Z0 (Figure 3d), exhibited a discontinuous morphology, suggesting more pronounced phase heterogeneity than in the SP-based matrix. At 1 wt% (Figure 3e), interfacial discontinuities remained and the bright inorganic domains were unevenly distributed. At 5 wt% ZCu (Figure 3f), the bright inorganic domains were more numerous and broadly distributed, although the matrix remained morphologically heterogeneous.

Overall, the micrographs indicate that the botanical starch source influenced the continuity of the compatibilized TPS/PLA matrix, while ZCu concentration mainly affected the distribution and local aggregation of the inorganic phase.

### 3.3. Thermal Properties

#### 3.3.1. Thermal Stability and Degradation Behavior by TGA

Thermal stability of the composites was evaluated by TGA, showing a formulation-dependent effect of ZCu incorporation. For the SP-based systems, the addition of 1 wt% zeolite increased thermal resistance with T_5%_ shifting from 132.94 °C (BM-SP/Z0) to 185.10 °C (BM-SP/Z1) as shown in Table 2. This improvement can be attributed to the well-dispersed zeolite domains acting as thermal barriers, limiting volatile diffusion and delaying the onset of decomposition. Additionally, interfacial interactions between the polymer matrix and the inorganic phase may restrict chain mobility, contributing to enhanced thermal resistance. This behavior is consistent with the relatively broad filler distribution observed by SEM and the crystallinity trend obtained by XRD, as well as with the inherent thermal stability of the zeolite structure [25].

**Figure 4:**
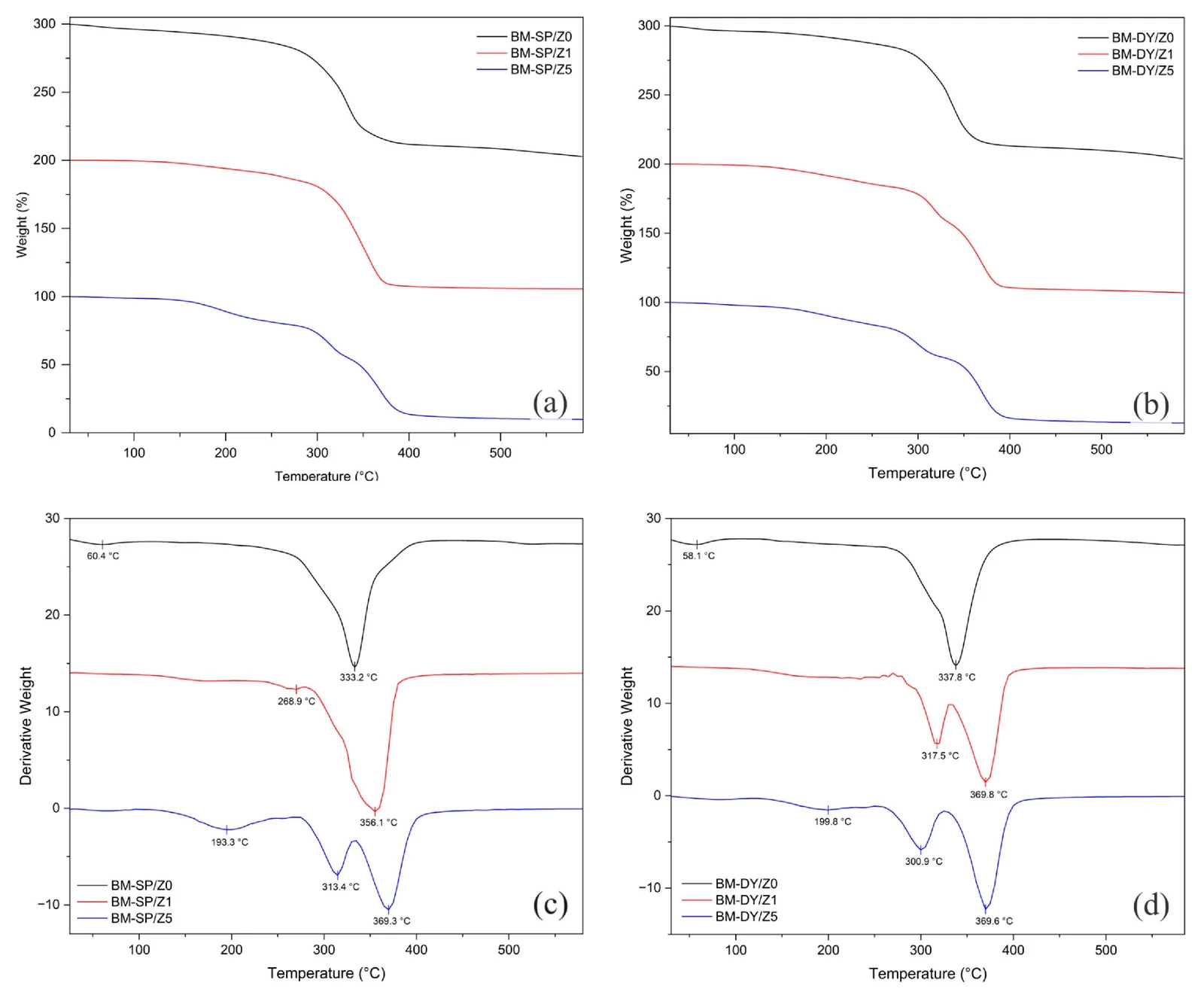
Thermograms (**a**,**b**) and derivative thermograms (**c**,**d**) of SP- and DY-based blends, respectively.

At 5 wt%, three degradation events were observed at 193.3, 313.4 and 369.3 °C for the SP-based composite and 199.8, 300.9 and 369.6 °C for the DY-based composite (Figure 4). The first event was tentatively associated with the early degradation of plasticizer-rich matrix fractions, whereas the subsequent events were assigned to the degradation of the TPS-rich and PLA-rich fractions, respectively [14,26,27]. The multi-step profiles indicate a more heterogeneous thermal response at the highest ZCu concentration.

Conversely, DY-based blends exhibited a more moderate stabilization response. Although T5% increases compared to the unfilled matrix, further loading to 5 wt% results in a slight reduction (164.81 °C). Unlike the SP-based system, the DY-based composites showed a smaller improvement in degradation onset temperature after ZCu incorporation, indicating a less pronounced thermal stabilization effect.

Notably, residue content increases progressively with reinforcement loading in both systems (Table 2), confirming the increasing contribution of the inorganic phase. However, this does not directly translate into improved thermal stability, as higher filler contents may promote particle aggregation and reduce dispersion efficiency, leading to non-uniform thermal barrier effects depending on the starch source. Overall, the largest increase in T5% was observed for BM-SP/Z1, whereas the DY-based composites exhibited smaller changes in degradation onset temperature and a higher residue content at 5 wt% ZCu.

#### 3.3.2. Thermal Transitions and Crystallization Behavior by DSC

DSC analysis provided complementary information on the crystallization behavior observed by XRD. Glass transition temperatures (T_g_) remain relatively stable across formulations as shown in Table 3, indicating that zeolite incorporation in some cases slightly increases or decreases the plasticization in the matrix [28].

**Figure 5:**
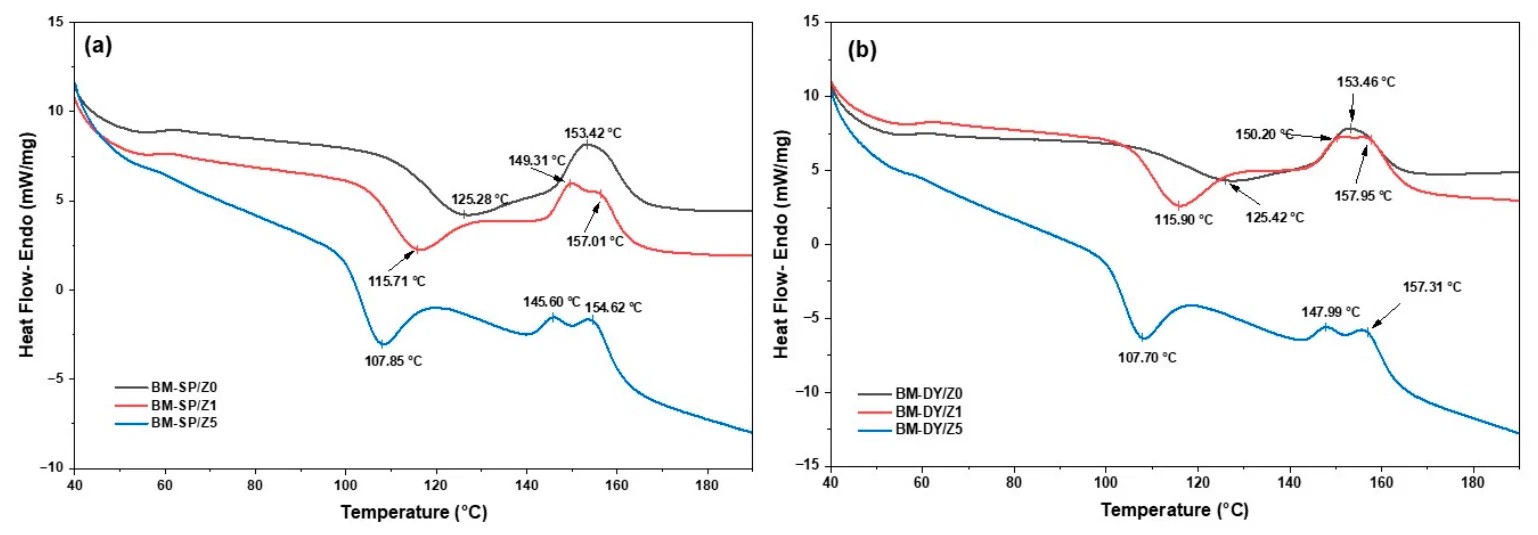
DSC thermograms of (**a**) SP- and (**b**) DY-based blends.

As shown in Figure 5, in SP-based systems, cold crystallization temperature (T_c_) decreases as the reinforcement loading increases, demonstrating that zeolite facilitates earlier crystallization upon heating. T_c_ was reduced by approximately 14% at the highest zeolite loading compared with the unreinforced system. Pires et al. reported that a reduction in T_c_ has been widely associated with improved heterogeneous nucleation efficiency of PLA induced by the inorganic phase; however, the possible contribution of additional factors cannot be excluded, including the formation of lower-molecular-weight chains or secondary polymeric species that may also promote crystallization at reduced temperatures [29].

The increase in ΔH_c_ suggests enhanced crystallization during heating, although the resulting crystalline phase may exhibit lower structural regularity. In SP-based systems, ΔH_c_ increased by up to 44% relative to the unreinforced material. The combined melting enthalpy remained lower in the reinforced SP-based formulations than in BM-SP/Z0, suggesting that the crystals formed after ZCu incorporation may exhibit lower structural perfection [30]. This trend is consistent with the highest estimated crystallinity observed for BM-SP/Z1.

In DY-based systems, a similar reduction in Tc is observed, but the enthalpic evolution differs. At 1 wt%, ΔH_c_ increases considerably despite reduced crystallinity in XRD, suggesting that crystallization kinetics are altered but structural reorganization remains constrained. Only at 5 wt% does the thermal behavior align with enhanced long-range ordering, consistent with the crystallinity increase to 12.75%.

The lower-temperature peak was associated with less perfect or thinner crystals, followed by melt-recrystallization or reorganization into more stable crystals that melted at the higher temperature. The coexistence of crystal populations with different lamellar perfection may also contribute [31]. Overall, DSC suggested that ZCu primarily affected crystallization behavior rather than T_g_. Although Tc decreased with increasing ZCu loading in both matrices, ΔH_c_ reached its highest value at 5 wt% ZCu in the SP-based system and at 1 wt% ZCu in the DY-based system, indicating a starch-source-dependent crystallization response.

### 3.4. Nanomechanical Characterization by Nanoindentation

**Figure 6:**
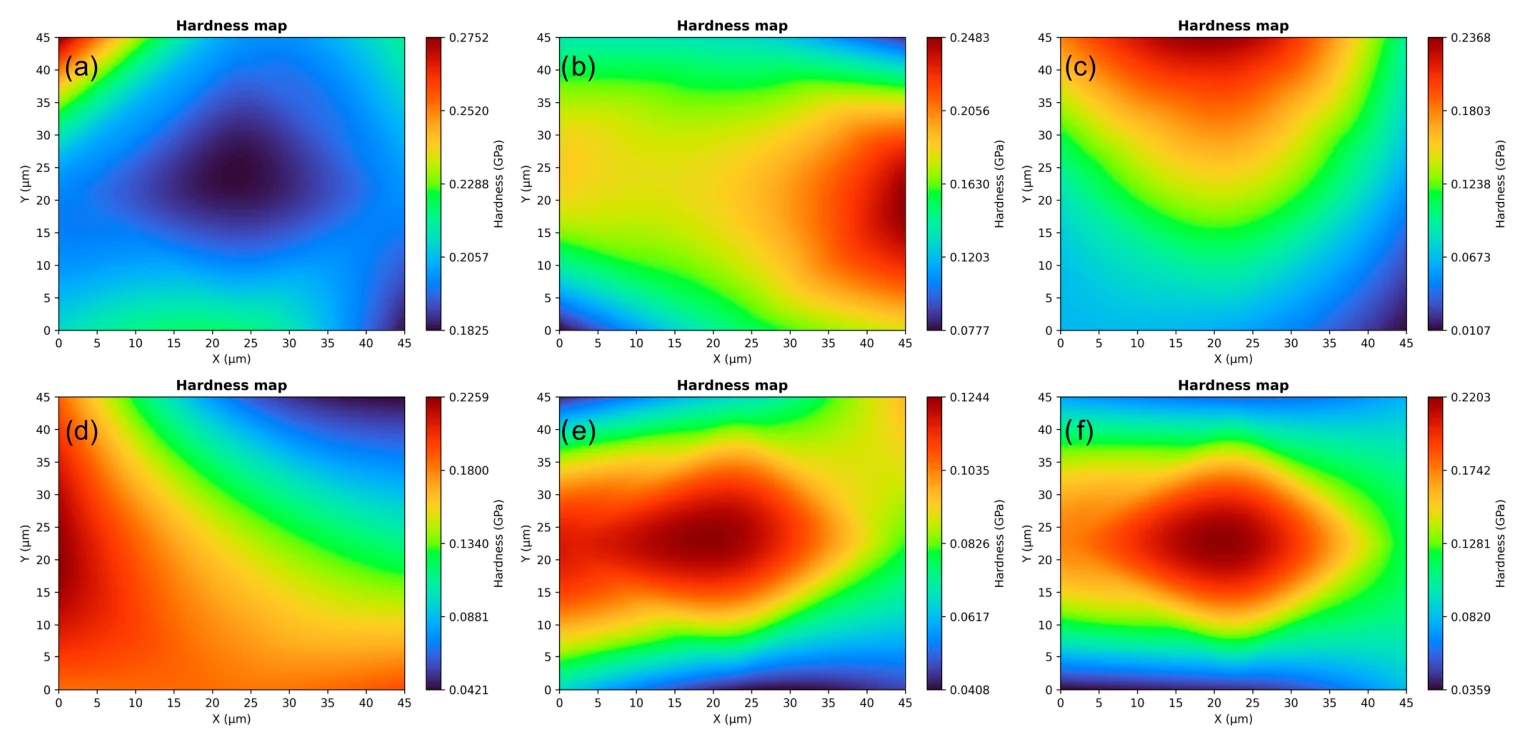
Local hardness distribution obtained by nanoindentation mapping for: (**a**) BM-SP/Z0, (**b**) BM-SP/Z1, (**c**) BM-SP/Z5, (**d**) BM-DY/Z0, (**e**) BM-DY/Z1, and (**f**) BM-DY/Z5.

Figure 6 reveals a pronounced dependence of the local hardness distribution on both the botanical origin of the starch and ZCu concentration, highlighting distinct nanomechanical responses between the SP- and DY-based systems. In the SP-based formulations (Figure 6a–c), BM-SP/Z0 exhibits hardness values ranging from approximately 0.183 to 0.275 GPa, with moderate spatial heterogeneity characterized by a localized softer region near the center of the mapped area. Upon incorporation of 1 wt% ZCu, BM-SP/Z1 displays a broader hardness range of approximately 0.078–0.248 GPa and a pronounced spatial gradient, with higher hardness values concentrated toward the right side of the mapped region. At 5 wt% ZCu, BM-SP/Z5 exhibits an even wider hardness distribution, ranging from approximately 0.011 to 0.237 GPa, with a relatively hard upper region and a markedly softer lower region. Therefore, ZCu incorporation into the SP-based matrix does not produce a monotonic increase in hardness; rather, it promotes increasingly heterogeneous local mechanical responses. This behavior suggests that the effect of ZCu is strongly influenced by its spatial distribution and by filler-induced changes in the local organization of the polymer matrix, consistent with the localized particle clustering observed by SEM at the highest ZCu concentration.

In the DY-based formulations (Figure 6d–f), a non-monotonic hardness response is observed. BM-DY/Z0 exhibits hardness values ranging from approximately 0.042 to 0.226 GPa, whereas the addition of 1 wt% ZCu reduces the hardness range to approximately 0.041–0.124 GPa, with a localized central region of comparatively higher hardness surrounded by softer regions. At 5 wt% ZCu, the maximum hardness increases again to approximately 0.220 GPa, and a broader, more symmetric central high-hardness region develops. Interestingly, this non-monotonic evolution qualitatively follows the crystallinity trend determined by XRD for the DY-based formulations, which decreases from 10.73% for BM-DY/Z0 to 9.94% for BM-DY/Z1 and subsequently increases to 12.75% for BM-DY/Z5, although the present results do not establish a direct causal relationship between crystallinity and hardness.

Overall, the hardness maps demonstrate that ZCu incorporation does not result in uniform strengthening of the TPS/PLA matrices. Instead, the local resistance to deformation appears to arise from the coupled effects of starch botanical origin, matrix organization, filler dispersion, and concentration-dependent matrix–filler interactions, leading to distinct spatial hardness distributions in the SP- and DY-based systems.

**Figure 7:**
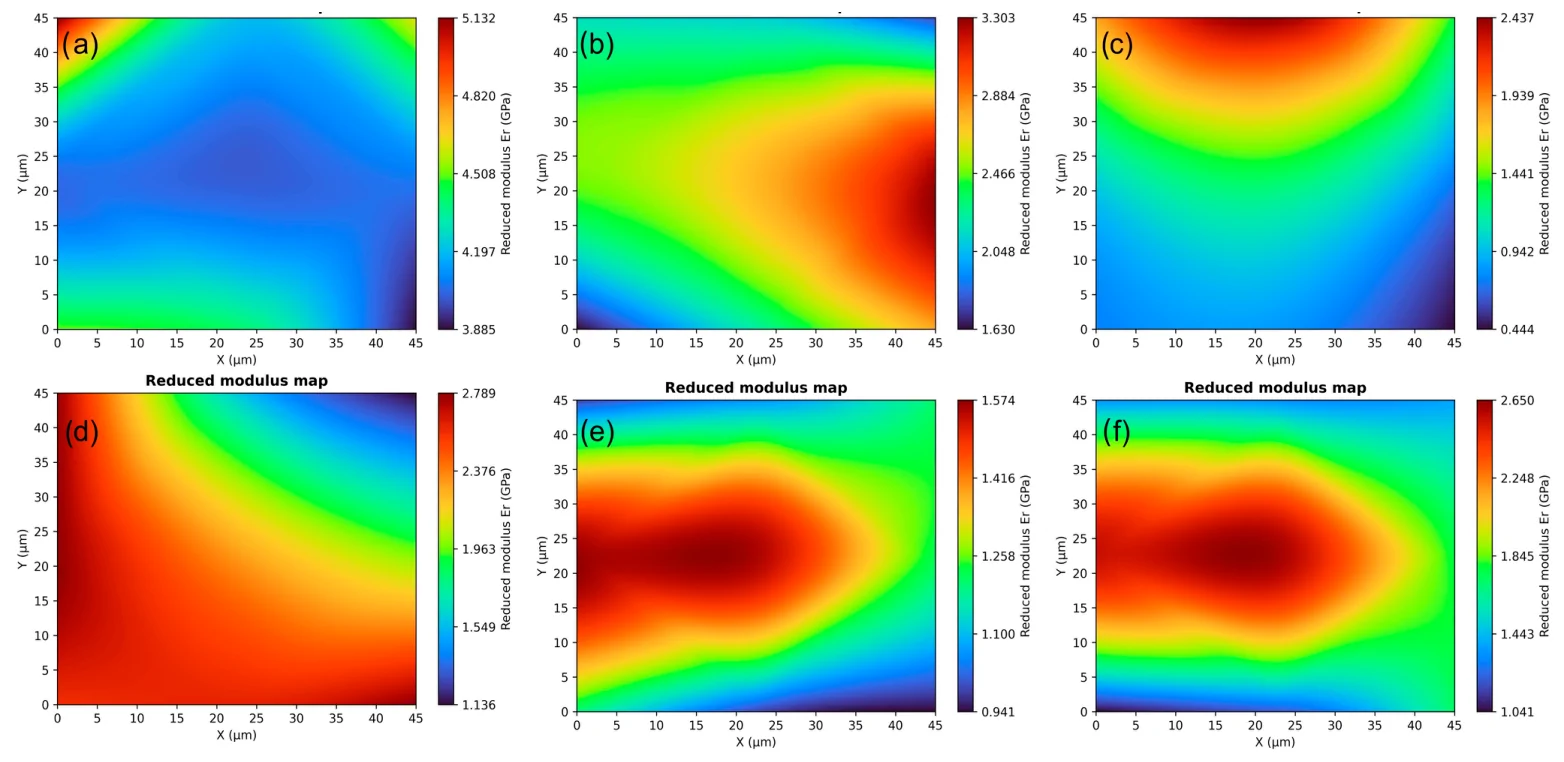
Spatial reduced modulus maps obtained by nanoindentation for: (**a**) BM-SP/Z0, (**b**) BM-SP/Z1, (**c**) BM-SP/Z5, (**d**) BM-DY/Z0, (**e**) BM-DY/Z1, and (**f**) BM-DY/Z5.

Figure 7 presents the spatial distribution of the reduced modulus obtained from nanoindentation mapping of the analyzed blends, providing complementary information to the hardness maps by describing the local elastic stiffness of the materials. Unlike hardness, which is primarily associated with resistance to irreversible deformation, the reduced modulus provides insight into the local elastic response and its spatial heterogeneity. In the SP-based formulations (Figure 7a–c), BM-SP/Z0 exhibits the highest reduced modulus, ranging from approximately 3.99 to 5.13 GPa, together with a comparatively homogeneous stiffness distribution. Upon ZCu incorporation, the reduced modulus progressively decreases, reaching approximately 1.63–3.30 GPa for BM-SP/Z1 and 0.44–2.44 GPa for BM-SP/Z5, while increasingly pronounced spatial gradients develop across the mapped regions. Therefore, ZCu incorporation into the SP-based matrix does not result in the conventional stiffening expected from the addition of a rigid inorganic filler; instead, the results indicate a reduction in local elastic stiffness accompanied by increased mechanical heterogeneity. This behavior may be associated with changes in the local organization of the polymer matrix and filler distribution, particularly considering the localized ZCu clustering observed by SEM at 5 wt%.

In the DY-based formulations (Figure 7d–f), a non-monotonic response is observed. BM-DY/Z0 exhibits reduced modulus values ranging from approximately 1.14 to 2.79 GPa, whereas the addition of 1 wt% ZCu decreases the modulus to approximately 0.94–1.57 GPa. At 5 wt% ZCu, the reduced modulus increases again, reaching values of up to approximately 2.65 GPa and exhibiting a broader central region of relatively high stiffness. Interestingly, this non-monotonic evolution qualitatively follows the crystallinity trend determined by XRD for the DY-based formulations, which decreases from 10.73% for BM-DY/Z0 to 9.94% for BM-DY/Z1 and subsequently increases to 12.75% for BM-DY/Z5, although a direct causal relationship between crystallinity and reduced modulus cannot be established from these measurements alone.

Overall, the reduced modulus maps demonstrate that ZCu does not produce uniform elastic reinforcement of the TPS/PLA matrices. Instead, the local stiffness response appears to result from the combined effects of starch botanical origin, matrix organization, filler dispersion, and concentration-dependent matrix–filler interactions, leading to distinct mechanical responses in the SP- and DY-based systems.

**Figure 8:**
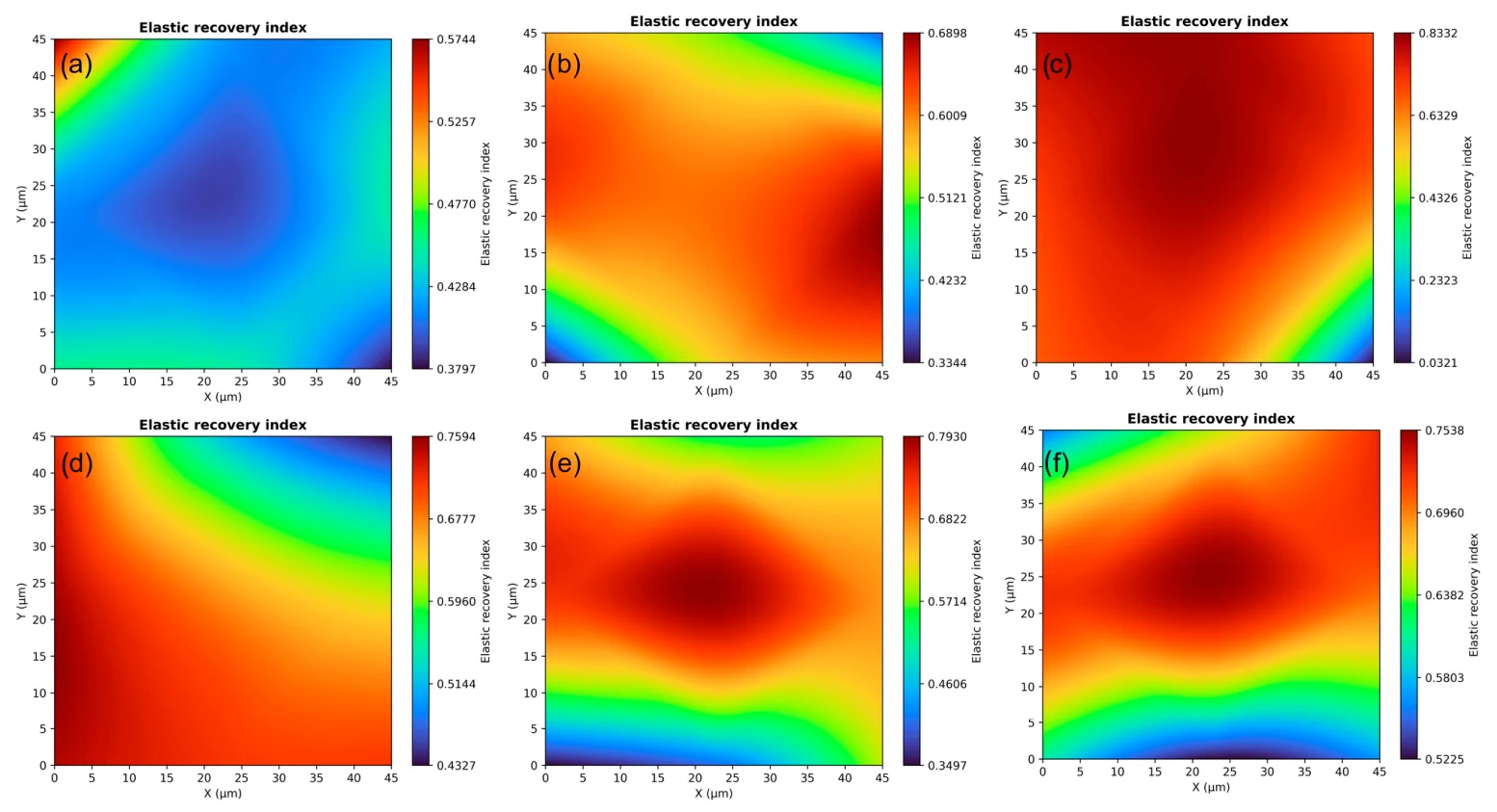
Spatial Elastic recovery index maps obtained by nanoindentation for: (**a**) BM-SP/Z0, (**b**) BM-SP/Z1, (**c**) BM-SP/Z5, (**d**) BM-DY/Z0, (**e**) BM-DY/Z1, and (**f**) BM-DY/Z5.

Elastic recovery maps reveal a clear dependence of the nanomechanical response on the botanical origin of the starch, indicating that this behavior cannot be attributed exclusively to ZCu concentration. In the SP-based system (see Figure 8a–c), elastic recovery progressively increases with ZCu incorporation, with BM-SP/Z0 exhibiting the lowest values and BM-SP/Z5 the highest. This behavior suggests that incorporation of the inorganic phase promotes a greater recoverable contribution to the indentation-induced deformation, possibly through local restriction of polymer-chain mobility and enhanced mechanical confinement of the matrix.

This interpretation is consistent with the comparatively more homogeneous morphology observed for the SP-based matrix and the distribution of ZCu domains, although some degree of particle clustering becomes evident at 5 wt%. In contrast, the DY-based formulations (see Figure 8d–f) exhibit relatively high elastic recovery values even in the unfilled BM-DY/Z0 matrix and display a non-monotonic response to ZCu incorporation, indicating that the intrinsic organization of the DY-based TPS/PLA matrix plays a significant role in its recovery behavior. The greater morphological heterogeneity observed by SEM in the DY-based formulations, together with their distinct crystallization response, suggests that ZCu incorporation produces competing effects between local restriction of polymer-chain mobility and the mechanical heterogeneity introduced by the inorganic phase and its interfaces.

Consequently, whereas ZCu incorporation progressively enhances elastic recovery in the SP-based system, this effect in the DY-based matrix is more strongly dependent on the local matrix–filler organization and does not exhibit a direct relationship with ZCu concentration. Overall, these results indicate that the elastic recovery of TPS/PLA–ZCu composites is governed by the coupled effects of starch botanical origin, matrix organization, filler dispersion, and interfacial constraints, rather than by the presence of the rigid inorganic phase alone.

It should be emphasized that nanoindentation characterizes the local contact response of the material at the micrometer scale. Therefore, the reduced modulus reported in this study should not be interpreted as the macroscopic tensile Young’s modulus of the composites. Likewise, nanoindentation does not provide direct information on tensile strength, elongation at break, impact strength, or overall structural toughness. Consequently, although the present results provide valuable insight into local nanomechanical behavior and its spatial heterogeneity, additional macroscopic mechanical characterization, together with barrier and processing assessments, is required before these composites can be validated for specific packaging applications.

### 3.5. Antimicrobial Performance of Zeolite-Reinforced Composites

**Figure 9:**
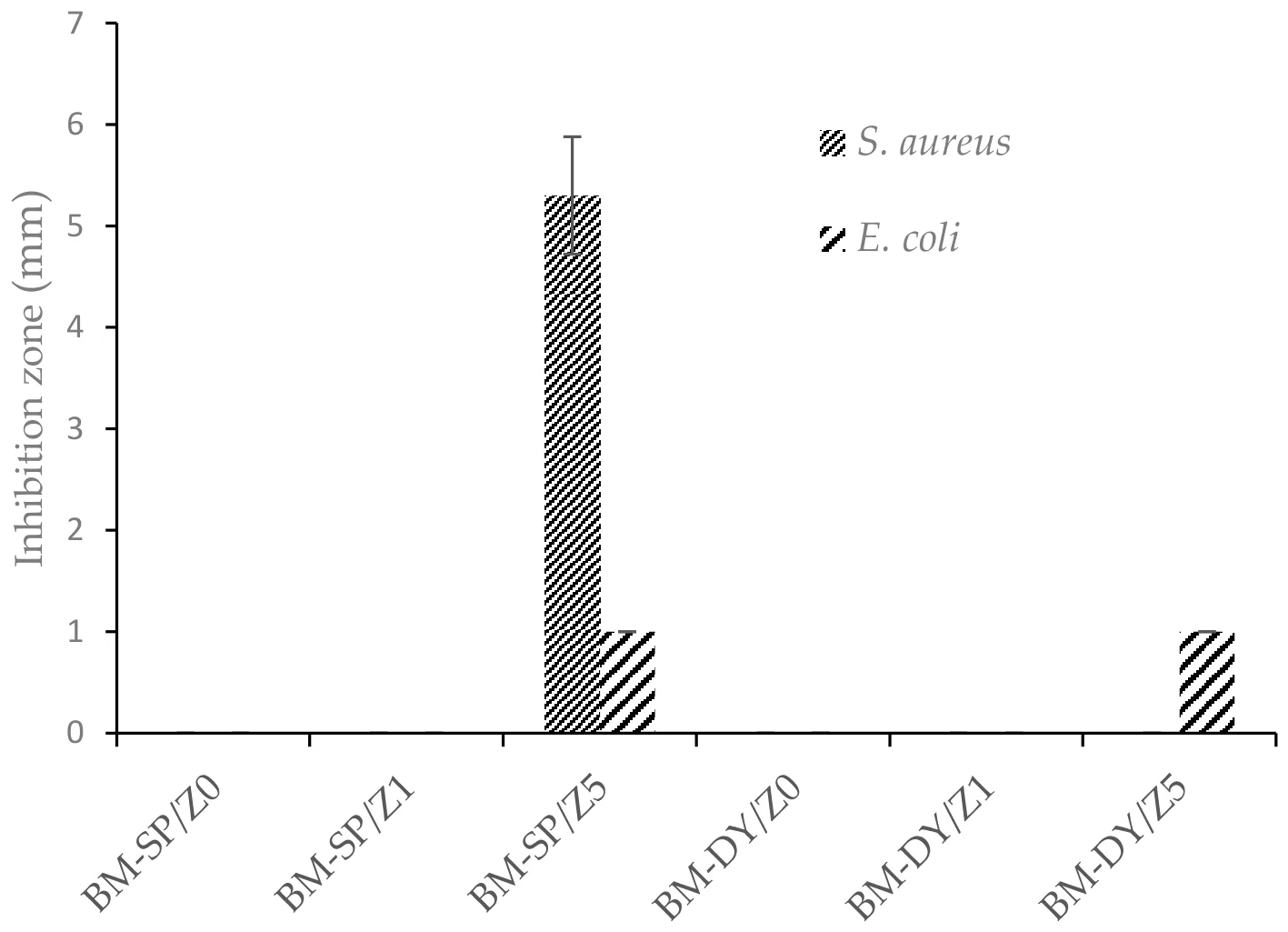
Zone of inhibition of *Escherichia coli* and *Staphylococcus aureus* exposed to different samples.

The antibacterial activity of SP-based systems and DY-based systems, with and without copper content, was evaluated using the zone of inhibition method, which was performed against a Gram-positive bacterium (*Staphylococcus aureus*) and a Gram-negative bacterium (*Escherichia coli*). As shown in Figure 9, the samples containing *Dioscorea rotundata* or *Ipomoea batatas* with 0% and 1% by weight concentrations of copper zeolite did not show an inhibition zone against the bacteria strains evaluated. The absence of inhibition zones at 1 wt% copper zeolite may be attributed to the low amount of copper available for release from the polymer matrix. Considering the nominal Cu loading of copper zeolite (8 wt%), the incorporation of 1 and 5 wt% copper zeolite corresponds to theoretical Cu contents of approximately 0.08 and 0.40 wt%, respectively. At 1 wt% copper zeolite, the amount of Cu species released and subsequently diffused through the polymer matrix into the agar may therefore remain below the threshold required to produce a detectable inhibition zone. In contrast, the samples containing 5% by weight of copper zeolite showed an inhibition zone.

The BM-SP/Z5 sample exhibited the greatest inhibition against *S. aureus*, yielding a zone of 5.3 mm; against that same strain, the BM-DY/Z5 sample showed no inhibition zone. For the *E. coli* strain, the BM-SP/Z5 and BM-DY/Z5 samples exhibited an inhibition zone of 1 mm. The results suggest that samples with a higher copper-zeolite content exhibit greater antibacterial activity, which may be due to increased release of copper ions. In this context, only the materials containing 5% copper zeolite exhibited antibacterial activity. As the results show, the largest inhibition zone was observed against *S. aureus*. In the case of *E. coli*, both materials formulated with 5% copper zeolite were able to inhibit bacterial growth.

The antibacterial effect of materials containing copper zeolite is primarily attributed to the action of the released copper ions. As reported by Nekooei-Fard et al., these ions interfere with cellular nutrient pathways, affecting essential metabolic processes and limiting microbial proliferation [32]. Complementarily, Huenuvil-Pacheco et al. described that Cu ions can bind to the cell membrane and bacterial cell wall through electrostatic interactions, causing structural alterations and damage to intracellular proteins [7]. Furthermore, it has been reported that copper induces significant changes in the lipid composition of proteins and nucleic acids through the generation of reactive oxygen species (ROS), compromising cellular integrity and viability [33].

## 4. Conclusions

The incorporation of copper-modified ZSM-5 zeolite influenced the structural, thermal, nanomechanical, and antimicrobial performance of compatibilized TPS/PLA composites, with the observed trends depending on both ZCu concentration and botanical starch source. Structural analyses demonstrated distinct matrix–filler interaction mechanisms for each starch type. In SP-based systems, BM-SP/Z1 exhibited the highest estimated crystallinity and a relatively broad distribution of the inorganic domains, whereas BM-SP/Z5 showed localized particle clusters. In the DY-based system, crystallinity initially decreased at 1 wt% ZCu and subsequently increased at 5 wt%, while the matrix remained morphologically heterogeneous.

TGA showed that the largest increase in degradation onset temperature occurred for BM-SP/Z1, whereas the DY-based composites exhibited a more moderate stabilization response and a higher residue content at 5 wt% ZCu. DSC indicated that ZCu primarily affected crystallization behavior rather than Tg. Although Tc decreased with increasing ZCu content in both matrices, the enthalpic responses differed according to starch source.

Nanoindentation mapping revealed that the incorporation of ZCu produced concentration- and starch-source-dependent local nanomechanical responses rather than uniform mechanical reinforcement. The hardness, reduced modulus, and elastic recovery distributions varied according to the botanical origin of the starch, filler concentration, matrix organization, and ZCu dispersion. In particular, the reduced modulus did not exhibit a monotonic increase with increasing ZCu content, indicating that the local mechanical response results from the combined effects of matrix–filler interactions and microstructural heterogeneity rather than from the presence of the rigid inorganic phase alone.

Antimicrobial activity was observed only for composites containing 5 wt% ZCu, indicating that the highest ZCu loading evaluated was required to achieve detectable antibacterial performance, particularly against Staphylococcus aureus. Overall, the effect of ZCu depended on both its concentration and the botanical origin of the starch, leading to trade-offs among structural organization, thermal stability, local nanomechanical behavior, and antibacterial activity. The SP-based composite containing 1 wt% ZCu exhibited the greatest improvement in thermal stability, whereas detectable antibacterial activity required 5 wt% ZCu. Because the nanomechanical characterization performed in this work was based on instrumented nanoindentation, the reduced modulus represents a local contact property and should not be interpreted as the macroscopic tensile Young’s modulus of the composites. Tensile strength, elongation at break, and impact strength were not evaluated. Therefore, comprehensive macroscopic mechanical, barrier, and processing evaluations remain necessary before these composites can be validated for specific packaging or manufacturing applications.

## Figures and Tables

**Table 1:** Crystallinity of the composites.

Composite	Crystallinity (%)
BM-SP/Z0	12.09
BM-SP/Z1	13.70
BM-SP/Z5	13.07
BM-DY/Z0	10.73
BM-DY/Z1	9.94
BM-DY/Z5	12.75

**Table 2:** TGA parameters of the composites.

Composite	T_5%_ (°C)	T_10%_ (°C)	T_d1_ (°C)	T_d2_ (°C)	T_d3_ (°C)	Residue (%)
BM-SP/Z0	132.94	215.14	60.4	-	333.2	2.80
BM-SP/Z1	185.10	245.10	-	268.9	356.1	5.65
BM-SP/Z5	169.81	194.80	193.3	313.4	369.3	9.79
BM-DY/Z0	150.19	221.50	58.1	-	337.8	3.96
BM-DY/Z1	169.80	214.80	-	317.5	369.8	6.82
BM-DY/Z5	164.81	201.81	199.8	300.9	369.6	12.61

**Table 3:** DSC parameters of TPS/PLA composites.

Composite	T_g_ (°C)	T_c_ (°C)	ΔH_c_ (J/g)	T_m1_ (°C)	ΔH_m1_ (J/g)	T_m2_ (°C)	ΔH_m2_ (J/g)
BM-SP/Z0	58.52	125.28	6.66	153.42	6.17	-	-
BM-SP/Z1	58.81	115.71	9.03	149.31	1.18	157.01	0.82
BM-SP/Z5	56.16	107.85	9.56	145.60	0.70	154.62	2.24
BM-DY/Z0	58.71	125.42	4.99	153.46	8.28	-	-
BM-DY/Z1	59.36	115.90	10.68	150.20	0.96	157.95	0.91
BM-DY/Z5	57.65	107.70	9.36	147.99	0.90	157.31	1.92

## Data Availability

The authors confirm that the data supporting the findings of this study are available within the article. In addition, the datasets used and/or analyzed during the current study are available from the corresponding author on reasonable request.
